# Long-Term Posttraumatic Stress Following Accidental Injury in Children and Adolescents: Results of a 2–4-Year Follow-Up Study

**DOI:** 10.1007/s10880-019-09615-5

**Published:** 2019-03-28

**Authors:** Els P. M. van Meijel, Maj R. Gigengack, Eva Verlinden, Alida F. W. van der Steeg, J. Carel Goslings, Frank W. Bloemers, Jan S. K. Luitse, Frits Boer, Martha A. Grootenhuis, Ramón J. L. Lindauer

**Affiliations:** 1 Department of Child and Adolescent Psychiatry, Amsterdam UMC, University of Amsterdam, Meibergdreef 5, 1105 AZ Amsterdam, The Netherlands; 2 de Bascule, Academic Center for Child and Adolescent Psychiatry, Amsterdam, The Netherlands; 3 Pediatric Surgical Center of Amsterdam, Amsterdam UMC, Emma Children’s Hospital, University of Amsterdam & VU University, Amsterdam, The Netherlands; 4 Trauma Unit Department of Surgery, Amsterdam UMC, University of Amsterdam, Amsterdam, The Netherlands; 5 Department of Trauma Surgery, Amsterdam UMC, VU University, Amsterdam, The Netherlands; 6 Emergency Department, Amsterdam UMC, University of Amsterdam, Amsterdam, The Netherlands; 7 Pediatric Psychology Department of the Emma Children’s Hospital, Amsterdam UMC, University of Amsterdam, Amsterdam, The Netherlands; 8 Princess Maxima Center for Pediatric Oncology, Wilhelmina Children’s Hospital, Utrecht, The Netherlands

**Keywords:** Child, Adolescent, Posttraumatic stress disorder (PTSD), Accidental injury, Long term

## Abstract

In this study, we determined the long-term prevalence of posttraumatic stress disorder (PTSD) in children and adolescents after accidental injury and gained insight into factors that may be associated with the occurrence of PTSD. In a prospective longitudinal study, we assessed diagnosed PTSD and clinically significant self-reported posttraumatic stress symptoms (PTSS) in 90 children (11–22 years of age, 60% boys), 2–4 years after their accident (mean number of months 32.9, SD 6.6). The outcome was compared to the first assessment 3 months after the accident in 147 children, 8–18 years of age. The prevalence of PTSD was 11.6% at first assessment and 11.4% at follow-up. Children with PTSD or PTSS reported significantly more permanent physical impairment than children without. Children who completed psychotherapy had no symptoms or low levels of symptoms at follow-up. Given the long-term prevalence of PTSD in children following accidents, we recommend systematic monitoring of injured children. The role of possible associated factors in long-term PTSS needs further study.

## Introduction

Accidents such as traffic accidents, sports accidents and falls are a major cause of pediatric unintentional injury (Brosbe et al. 2011; Kassam-Adams et al. 2013; van Meijel et al. 2015). Besides physical injuries, children can suffer from posttraumatic stress symptoms following accidents. The majority of the children recover within a few weeks, but if the symptoms persist for more than 1 month and cause significant impairment in one or more areas of functioning, posttraumatic stress disorder (PTSD) can be diagnosed. PTSD is a debilitating psychiatric disorder. If left untreated, PTSD negatively affects children’s functioning and physical recovery from injury (Kahana et al. 2006; Kassam-Adams et al. 2013). Evidence-based psychological treatments for PTSD are available and have proven to be effective in children with multiple types of trauma (Cohen et al. 2010; Morina et al. 2016; Smith et al. 2018). However, it is likely that PTSD in many children and adolescents remains undiagnosed and that not everyone with a PTSD diagnosis receives adequate trauma-focused therapy (Mehta and Ameratunga 2012; National Institute for Clinical Excellence (NICE) 2005; Smith et al. 2018). Without treatment, symptoms can be prolonged or worsen significantly over time. Moreover, they are often associated with other severe, long-term effects such as psychosocial problems and learning difficulties (NICE 2005).

Although the long-term impact of traumatic events can be substantial, research on the long-term psychological consequences of accidental injury is scarce. Regarding long-term prevalence of posttraumatic stress reactions, we found only one recent study that assessed posttraumatic stress reactions following accidents beyond two years (Arnberg et al. 2011). This study examined seven survivors of a bus crash—all 12-year-old schoolchildren—with multiple injuries. This group still reported posttraumatic stress symptoms, such as sadness, feelings of guilt, intrusions and avoidance, 20 years after the accident. They reported significantly more symptoms than a group of 33 indirectly affected persons. The findings of this study suggest that traumatic accidents are associated with long-term posttraumatic stress reactions, but the limitations of the small sample and lack of representativeness on age preclude further conclusions.

Other studies had a follow-up period of 2 years or less after an accident (Alisic et al. 2014; Brosbe et al. 2011; Gillies et al. 2003; Hiller et al. 2016; Olofsson et al. 2009). In their meta-analysis, Alisic et al. (2014) found a prevalence of 9.7% PTSD for non-interpersonal trauma, including accidents. In this meta-analysis, PTSD was assessed by clinical interview and the age range was 2–18 years. In a follow-up study of road accident victims (aged 6 to 20 years) that was conducted up to 18 months after the accident, Gillies et al. (2003) found that 19% of the participants had ongoing problems with physical injury. Measured by child self-report, 34% of the children had continuing or delayed onset symptoms of PTSD. In a literature review to determine the prevalence of PTSD among 5–18-year-old children and adolescents injured in traffic, Olofsson et al. (2009) reported a prevalence of 13% at 3–6 months after the accident. PTSD in the included studies was assessed by diagnostic interview and/or self-report. They included only one study with a 2–18-month follow-up of victims of motor vehicle accidents, which reported 14% PTSD. In a meta-analytic study on changes in the prevalence of child PTSD in the year following trauma, the prevalence decreased from 21% in the acute phase to 11% after 1 year (Hiller et al. 2016). The majority of the studies included in this meta-analytic study focused on accidental injury and non-intentional trauma exposure in children 5–18 years old. Measurement of PTSD was done by self-report with a cut-off value, or by diagnostic interview.

Previous studies suggest that physical impairment, psychosocial consequences, trauma history, new traumatic events and trauma-focused psychotherapy are associated with the occurrence of long-term PTSD (Copeland et al. 2007; Gillies et al. 2003; Janssens et al. 2009; Landolt et al. 2005; Mehta and Ameratunga 2012; NICE 2005; Zatzick et al. 2008). Pain after accidental injury contributes to later PTSD or PTSS in children and adolescents (Hildenbrand et al. 2016); in particular, severe acute pain is associated with PTSS 3 months later (van Meijel et al. 2018). The long-term effects of acute pain in accidentally injured children have not been reported as yet.

Although non-injured or mildly injured children can also develop PTSD (Olofsson et al. 2009), serious injury with long-term physical impairment as a consequence may be associated with long-term health and mental health problems. A long-term follow-up study in children 7 years after major trauma revealed that about 40% of the children were physically impaired and half of this group was restricted in daily activities (Janssens et al. 2009). Gillies and colleagues suggested that continuing physical problems may contribute to ongoing psychological distress (Gillies et al. 2003).

Zatzick and colleagues found an association between high levels of recurrent traumatic life events before the injury and PTSD in injured adolescents 12 months after the accident (Zatzick et al. 2008). Additionally, they suggested that traumatized adolescents are at risk for recurrent posttraumatic life events, including reinjury. PTSD and comorbid disorders (e.g. depression) have been shown to have a negative effect on social relationships, which can lead to social withdrawal, break up of significant relationships and problems in the family (Mehta and Ameratunga 2012; NICE 2005). In the general population of children, multiple trauma exposure results in posttraumatic stress symptoms after a next potentially traumatic event (Copeland et al. 2007).

Natural recovery of posttraumatic stress symptoms in children can be promoted and facilitated by mechanisms such as post-trauma social support and family cohesiveness (Kazak et al. 2005). These mechanisms can be seen as protective factors and may reduce the risk of persistent PTSS. Furthermore, the resilience of parents appears to play a key role in their children’s emotional recovery; children of resilient parents were most likely to be resilient themselves (Le Brocque et al. 2010). Early screening to identify parents and families that are in need of support in the acute stage following a child’s accident can expedite the recovery of children (Muscara et al. 2018). As indicated above, evidence-based psychological treatments, including trauma-focused psychotherapy, have proven to be effective for children with significant symptoms or chronic PTSD. The association between the long-term consequences of accidental injury and whether or not children have received adequate trauma-focused psychotherapy is still unknown.

If we could determine the long-term prevalence of PTSD in children following accidents and confirm identifying factors that are associated with the long-term occurrence of PTSD, this would provide valuable insight with regard to treatment efforts and prevention of long-term negative consequences for children injured in accidents.

The aims of the present study were twofold: (1) to measure the prevalence of PTSD in children and adolescents, 2–4 years after accidental injury compared with 3 months after the accident; (2) to gain insight into individual factors that are associated with the occurrence of PTSD at follow-up: permanent physical impairment, acute pain, trauma history and new traumatic events and trauma-focused psychotherapy between the first and follow-up assessment.

## Methods

### Participants and Procedures

For reasons of brevity and readability, we decided to use one term for the participants in this manuscript, instead of specifying various age groups of children, adolescents and young adults. Since parents were also involved in the study to report about their children, we considered it appropriate to use “parents and children”.

From 2008 to 2010, we conducted a study in which we evaluated the Screening Tool for Early Predictors of PTSD (STEPP; Winston et al. 2003), a screening instrument to determine the risk of PTSD in children who had been injured due to accidental trauma (van Meijel et al. 2015). The STEPP study concluded with the assessment of PTSD 3 months after the accident (T1). The follow-up assessment was not scheduled in the design of the initial study. In 2012, we had the opportunity to conduct a follow-up assessment but we were limited in time. Despite resulting variability due to the range of 2 to 4 years in follow-up, we decided to use this opportunity.

For the current follow-up study, we approached the families (the children and one of their parents) who had participated in the first study and we assessed child PTSD 2 to 4 years after the accident (T2). The families received a letter in which the follow-up study was announced, including an explanation of the purpose of the study. Subsequently, we contacted the families via telephone. They were invited to participate in a telephone interview and to complete one questionnaire sent by email. Consent was given either in writing (by email) or during the initial telephone conversation (in which case this part of the conversation was audiotaped). The current study was approved by the Medical Ethical Committees of both hospitals of the Amsterdam UMC in Amsterdam, the Netherlands, and was performed from October 2012 to March 2013.

**Fig. 1:**
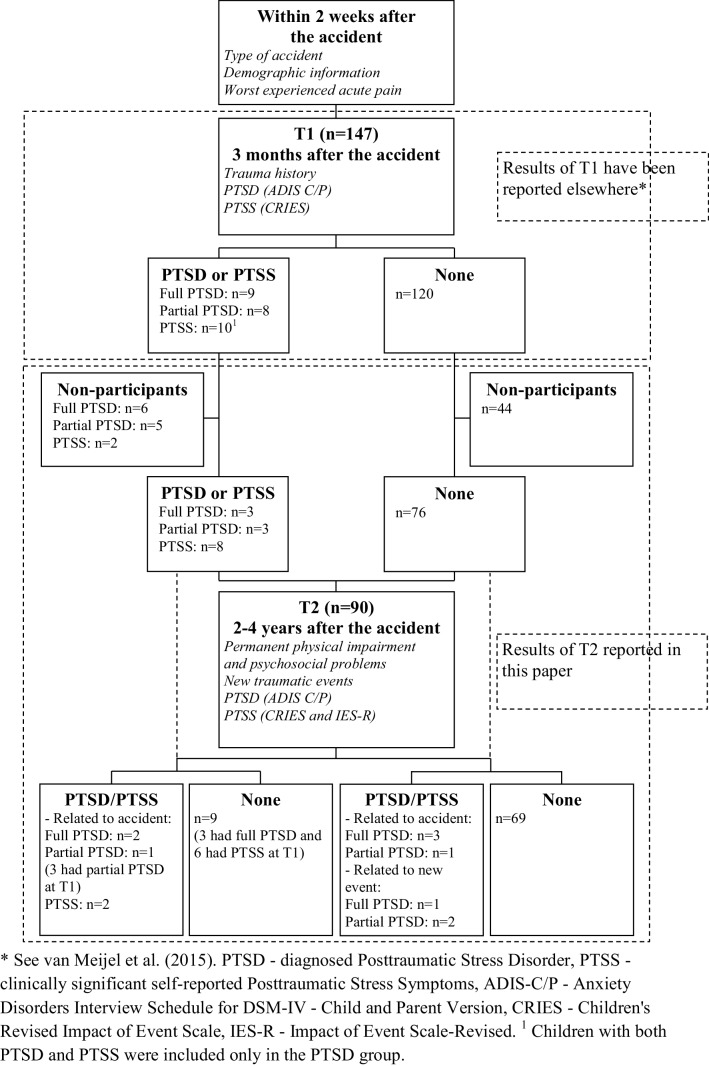
Flowchart of study participation, measures and PTSD/PTSS at T1 and T2. *See van Meijel et al. (2015). *PTSD* diagnosed posttraumatic stress disorder, *PTSS* clinically significant self-reported posttraumatic stress symptoms, *ADIS-C*/*P* Anxiety Disorders Interview Schedule for DSM-IV—Child and Parent Version, *CRIES* Children’s Revised Impact of Event Scale, *IES-R* impact of event scale-revised. ^1^Children with both PTSD and PTSS were included only in the PTSD group

Of the 147 participating families in the first study, 90 families (61%) participated in the follow-up study. See Fig. 1, Flowchart of participation. Of the initial group, 33 families could not be reached (4 telephone numbers were no longer in use and 29 did not answer the call) and 24 declined to participate. Reasons for declining participation were serious medical and/or psychological problems (3 families) and lack of time or no interest (21 families). Of this group of 90 participants, 62 (69%) had been involved in a traffic accident, 15 (17%) in a sports accident and 13 (14%) in other types of accidents, including falls.

The mean time between T1 and T2 was 32.9 months (SD = 6.6, range 22 to 49 months); the median was 33 months. In total, 54 boys (60%) and 36 girls (40%) participated at T2. Mean age of the children at T2 was 17.4 years (SD = 2.9, range 11 to 22 years). There were no significant differences between participants and non-participants with regard to age (*U* = 2564, *Z* = − 0.004, *p* = 0.99) or sex (*χ*^2^ = 0.064, *p* = 0.80). Follow-up participants reported significantly fewer posttraumatic stress symptoms at T1 than non-participants did (*U* = 1809, *Z* = − 2.628, *p* < 0.01).

### Measures

#### Demographic Information, Type of Accident, Acute Pain and Trauma History

Demographic information and information on the type of accident was obtained from the medical records shortly after the accident. Within two weeks after the accident, children reported the worst acute pain since the accident with the Visual Analogue Pain Scale (VAS). The VAS has a good reliability (intraclass correlation = 0.79), good correlation with the Faces Pain Scale-Revised scale (*r* = 0.72) and strong correlation with the Colour Analogue Scale (*r* = 0.92) (Le May et al. 2018). The VAS scores range from 0 to 10 and can be classified as *no or mild pain* (0–3), *moderate pain* (4–7), and *severe pain* (8–10). See van Meijel et al. (2018) for full details of pain assessment and the VAS. Trauma history from before the accident was assessed at T1.

#### Posttraumatic Stress Disorder

At both T1 and T2, diagnostic interviews were conducted with both the parent and child to determine the severity of PTSD symptoms in the children. In the Netherlands, the Dutch version of the Anxiety Disorders Interview Schedule for DSM-IV—Child and Parent Version (ADIS-C/P) is used to diagnose PTSD in children (Siebelink and Treffers 2001; Silverman and Albano 1996). The ADIS-C/P is a commonly used diagnostic, semi-structured interview for the assessment of anxiety disorders—including PTSD—and mood and behavioral disorders in children aged 7–17 years. The ADIS-C/P has a good to excellent test–retest reliability for specific diagnoses (*κ* = 0.61–1.00; Silverman et al. 2001) and inter-rater reliability (*κ* = 0.65–1.00; Lyneham et al. 2007). Although the ADIS C/P was not designed for young adults 17–22 years old, we used this interview because it is child and parent informed and because it enabled us to compare T1 and T2 results more effectively. Cronbach’s alphas were 0.84 for the child score and 0.77 for the parent score.

Depending on the answer and the clinical interpretation of the interviewer, symptoms can be rated as present or absent. If the number of symptoms endorsed as ‘yes’ is enough to meet DSM-IV-TR PTSD criteria (APA 2000), impairment in daily functioning is rated on a 9-point Likert scale (0–8). A diagnosis of PTSD requires an impairment level of 4 or more and depends also on the clinician’s judgment of clinical severity. The diagnosis can be based upon either the child report (C) or the parent report (P), or a combination of both reports. Partial PTSD is diagnosed when at least one symptom is present in each of three subscales—re-experiencing, avoidance and hyperarousal—resulting in substantial distress or impairment in one or more areas of functioning (Winston et al. 2003). The PTSD module of the ADIS C/P was administered with regard to the accident. If indicated it was also administered with regard to any new traumatic event that had happened between T1 and T2. In the present study, PTSD refers to diagnosed PTSD, including diagnosed partial PTSD.

#### Clinically Significant Self-reported Posttraumatic Stress (PTSS)

At T1, children completed the Dutch version of the Children’s Revised Impact of Event Scale (CRIES; Children and War Foundation 1998; Olff 2005; Verlinden et al. 2014). This self-report measure gives a good indication of the presence of PTSD. It consists of 13 questions in the subscales re-experiencing, avoidance and hyperarousal, with answers on a 4-point scale. Items are rated according to the frequency of their occurrence during the past week (not at all = 0, rarely = 1, sometimes = 3 and often = 5; range 0–65). We asked the children to focus on their accident when answering the questions. The cut-off score for a positive test is 30 (Verlinden et al. 2014). The outcome correlates highly with the PTSD diagnosis according to the Anxiety Disorders Interview Schedule for DSM-IV, Child and Parent Version (ADIS C/P; Verlinden et al. 2014). The CRIES has excellent test–retest reliability (*κ* = 0.85) and good reliability (Cronbach’s alpha = 0.89) (Verlinden et al. 2014). For the current sample Cronbach’s alpha = 0.91.

At T2, we used two self-report measures: one for children under 18 and one for children 18 years and older. The children under 18 completed the CRIES (see T1 above) and children 18 years and older completed the Dutch version of the Impact of Event Scale-Revised (IES-R; Horowitz et al. 1979; Weiss 2007). The IES-R consists of 22 questions and contains the subscales re-experiencing, avoidance and hyperarousal. Scoring is on a 5-point Likert scale. Items are rated according to the frequency of their occurrence during the past week (not at all = 0, a little bit = 1, moderately = 2, quite a bit = 3, extremely = 4; range 0–88). The focus is on the child’s accident. A total score of 23 or above indicates the likely presence of PTSD (Mouthaan et al. 2014). The Dutch IES-R showed adequate similarity with the total score of the Clinician-administered PTSD scale (CAPS; *r* = 0.75; *p* < 0.001) (Hovens et al. 1994; Mouthaan et al. 2014; Weathers et al. 2001) and good reliability for the current sample; Cronbach’s alpha = 0.93. In the present study PTSS (posttraumatic stress symptoms at a clinically significant level) refers to self-reported posttraumatic stress symptoms at a score of 30 or above (CRIES) or 23 or above (IES-R).

#### Health and Mental Health and New Traumatic Events

The follow-up interviews were composed by EM, MRG and RL and are available on request from the first author. Parents and children were interviewed separately by telephone. Parents were interviewed about their child. The interview started with the following open-ended questions: “How are things going? What has happened since we last met?” The purpose of this initial part of the interview was to become informed about the interviewee’s perception of the course of posttraumatic stress reactions over time and about any other relevant health and mental health-related information. We explicitly asked whether the child still experienced physical impairment and/or psychosocial consequences as a result of the accident. In our study, permanent physical impairment was defined as loss or abnormality of parts of the body, resulting in restrictions or inability to perform activities that were considered normal before the accident and are normal for children of that age. Examples of permanent physical impairment are chronic or frequent pain, walking with a limp and chronic fatigue. Besides physical impairment, details of psychosocial consequences of the accident (such as delay in school career, change of future plans, limitations in social life) were also assessed. A specific question was included regarding any new traumatic or life events: ‘Since the accident, have other stressful things happened to you?’ If necessary, we asked supplementary questions to assess whether an event was traumatic according to DSM-IV-TR criteria (APA 2000). If a child experienced one or more new traumatic events, we asked the child if help in any form was needed. If applicable, the choices regarding trauma-focused psychotherapy between T1 and T2 and its outcome were discussed.

#### PTSD

The second part of the interview consisted of the PTSD module of the ADIS-C/P (see the previous subsection “Posttraumatic Stress Disorder”).

Figure 1 provides an overview of measures used at the different time points.

### Statistical Analysis

Answers to questions on permanent physical impairment, psychosocial consequences and new traumatic events were classified by the first author and confirmed by the second author. Differences were discussed until consensus was reached. According to the definitions (see “Measures, health and mental health…”), answers were coded dichotomously: present ‘yes’ or ‘no’. Thereafter, we quantified the answers. The frequencies were used to compare the groups with and without PTSD or PTSS. Information on trauma-focused treatment between T1 and T2 was described in relation with PTSD or PTSS outcome at T2.

Differences between follow-up participants and non-participants were analyzed with Mann–Whitney *U* tests for age and posttraumatic stress at the time of the first assessment, and a Fisher’s exact test for sex. The statistical significance of differences between children with and without PTSD was determined with Mann–Whitney *U* tests for the mean acute pain scores and the number of traumatic events until follow-up and with Fisher’s exact test for the other items: the number of children (1) with trauma history before the accident (2) that experienced a new traumatic event between T1 and T2 (3) that reported severe acute pain and (4) with permanent physical impairment. Statistical significance was set at an alpha level of 0.05. Confidence intervals were calculated with CIA (Confidence Interval Analysis 2018). Other statistical analyses were performed using SPSS 24 (IBM Statistical Product and Service Solutions, Chicago, IL).

## Results

### Participants

In total, we included 90 children in this follow-up study. We interviewed 75 parents and 80 children at T2, resulting in interview-based data for 88 children. Of this latter group, 75 children completed the questionnaire and 73 also participated in the interview. The remaining two children completed the questionnaire but did not participate in the interview. In total, data on 90 children were available.

### The Prevalence of PTSD and PTSS at T1 and T2

At T1, 3 months after the accident, PTSD was diagnosed with the ADIS interview in 17 of 147 children (11.6%; 95% confidence interval (CI) 7.3–17.7%). The scores of 23 of 144 children (16%; 95% CI 10.9–22.8%) were above the cut-off score of the self-report measure CRIES. Of these children, 13 also received a PTSD diagnosis and 10 did not. At T2, the follow-up assessment, 10 of 88 children (11.4%; 95% CI 6.3–19.7%) were diagnosed with PTSD. On the self-report measures, the scores of eight of 75 children (10.7%; 95% CI 5.5–19.7%) were above the cut-off score, indicating the presence of PTSD. Of these children, six also received a PTSD diagnosis and two did not. At T2, in seven children, PTSD was related to the accident, and in three children it was related to a new event (sexual abuse, traumatic family circumstances and interpersonal violence, respectively). Moreover, two of these three children still suffered from substantial posttraumatic stress symptoms due to the accident. Figure 1 illustrates the course of participation of children with and without PTSD or PTSS from T1 up to and including T2.

### Factors Associated with the Occurrence of PTSD or PTSS at Follow-Up

#### Permanent Physical Impairment

At T2, children reported several types of permanent physical impairment as a consequence of the accident, such as chronic or frequent pain, disability of the back, a leg or a knee, walking with a limp, infertility, partial deafness, chronic fatigue, dysfunctioning of an eye and numbness of an arm, hand or fingers. In total, 27 of 88 children (31%; 95% CI 22–41%) reported permanent physical impairment; two of these children reported planned surgery due to ongoing physical problems. Of this group of 27 children, 9 had PTSD or self-reported PTSS and 18 did not.

Moreover, as a result of the accident and/or the permanent physical impairment, 23 of these 27 children were confronted with one or more major, primarily psychosocial, consequences. These included concentration problems due to headaches, delay in finishing a study program or dropping out, changing to lower level or type of education, serious limitations in participating in sports, inability to tolerate commotion or noise, inability to multitask, limitations in work or social life, no longer feeling at ease with peers, loss of friends and loneliness. Three children specifically mentioned a change of future plans due to physical limitations and chronic pain. These children had planned to become a professional athlete, a sports teacher and a plumber, respectively.

In the group with PTSD or PTSS at T2, a significantly higher percentage of children reported permanent physical impairment including psychosocial consequences, than the group without PTSD or PTSS. See Table 1 for more details and *p* values.

**Table 1:** Differences between children with and without PTSD or PTSS at T2

	Children with PTSD or PTSS	Children without PTSD or PTSS	Difference *p* value
*N* ^a^	12	78	
Sex—male	7 (58%)	47 (60%)	0.90^b^
Number of children with trauma history before the accident	10 (83%)	47 (60%)	0.32^b^
Number of children with new traumatic event between T1 and T2	4 (33%)	13 (17%)	0.24^b^
Mean number of traumatic events until T2 (SD, min–max)	3.6 (2.3, 1–10)	2.6 (1.8, 1–8)	0.06^c^
Mean acute pain score (SD, min–max)	8.0 (1.7, 5–10)	6.7 (2.5, 0.7–10)	0.12^c^
Number of children with severe acute pain	6^d^ (60%)	31^e^ (42.%)	0.32^b^
Number of children with permanent physical impairment	9 (75%)	18^f^ (24%)	0.001*^b^

*T2* at follow-up, *PTSD* diagnosed posttraumatic stress disorder, *PTSS* clinically significant self-reported posttraumatic stress symptoms, *SD* standard deviation

*Statistically significant difference between groups

^a^Children with both PTSD and PTSS were included only in the PTSD group

^b^Mann–Whitney *U* test was used

^c^Fisher’s exact test was used

^d^Pain ratings for two children were missing

^e^Pain ratings for four children were missing

^f^Information for two children was missing

#### Acute Pain

Acute pain scores of 84 children were available. In total, seven children reported no or mild pain, 40 children reported moderate pain, and 37 children reported severe pain. We found no significant difference between the groups with and without PTSD or PTSS at T2 regarding acute pain. See Table 1 for more details and *p* values.

#### Trauma History and New Traumatic Events Between T1 and T2

The mean number of traumatic events children experienced from before the accident until T2 was 3.6 in children with PTSD or PTSS and 2.6 in children without. Between T1 and T2, 16 children experienced one new traumatic event, and one child experienced two new traumatic events. The following traumatic events were reported: life-threatening intoxication, fire, sexual abuse, life-threatening illness of parent, severe (chronic) illness (3×), traffic accident (3×), life-threatening bleeding after surgery, interpersonal violence (2×), unknown (does not want to say), severe bullying over a long period of time, several suicide attempts of a friend, traumatic family circumstances and witnessing a severe traffic accident. There was no difference between the groups with and without PTSD or PTSS at T2 regarding trauma history before the accident or experiencing a new traumatic event between T1 and T2. More details and *p* values are provided in Table 1.

#### Psychological Treatment and Recovery in Follow-Up Participants

All three children who were diagnosed with full PTSD at T1 completed psychological trauma-focused therapy. One child received Eye Movement Desensitization and Reprocessing (EMDR); two others received Trauma-Focused Cognitive Behavioral Therapy (TF-CBT). At T2, they were fully recovered and reported no symptoms or low levels of symptoms. There was no indication from the interview that there were any other mental health problems. The three children who were diagnosed with partial PTSD at T1 were also advised to take trauma-focused therapy after the diagnosis was made. Two of the three children started therapy (one child EMDR, the other TF-CBT) but did not complete it; the third did not want to participate in psychological therapy. These three children still reported high levels of symptoms and were still diagnosed with PTSD at T2. In two of the children the partial PTSD developed into full PTSD between T1 and T2.

None of the eight children with self-reported PTSS at T1 received trauma-focused therapy; six children recovered spontaneously and two children still met criteria for self-reported PTSS at T2. Children who no longer fulfilled self-reported PTSS criteria at T2 retrospectively attributed the high score at T1 to stressful circumstances other than the accident.

## Discussion

The prevalence of PTSD at first assessment and at long-term follow-up was 11.6% and 11.4%, respectively. Our findings are consistent with those of Hiller et al. (2016). In their meta-analytic study, they reported a prevalence of 11% at 1 year after non-intentional trauma exposure. Compared to children without PTSD or PTSS, children with PTSD or PTSS reported significantly more permanent physical impairment. Our findings indicate that there may be an association between permanent physical impairment and long-term PTSD or PTSS but an association between the other individual factors and PTSD or PTSS is not indicated.

Although some of the children in our study recovered from PTSD following a successful trauma-focused therapy, in other children symptoms developed later on, continued at the initial level, or worsened from partial to full PTSD. Some children developed PTSD following new traumatic events, while still suffering from posttraumatic stress symptoms associated with the accident. The prevalence of PTSD at follow-up demonstrates the importance of being aware of the long-term consequences of accidents. It also indicates that long-term monitoring of children following accidents is appropriate, in line with the “best practice” following acute trauma, as proposed by the NICE (2005). The NICE guideline (NICE 2005) recommends “watchful waiting” including screening to identify those at risk who will benefit from further monitoring and timely therapeutic intervention. These recommendations could be applied in practice by implementing Trauma Informed Care (TIC), a multidisciplinary approach to reduce the risk for persisting posttraumatic stress and PTSD after injury (Marsac et al. 2016; Weiss et al. 2017). TIC uses trauma-related knowledge in medical practice, and can facilitate the implementation of a hospital monitoring system after injury, including timely interventions if needed. Our findings regarding self-reported PTSS and spontaneous recovery are in line with those of Verlinden et al. (2014), who showed that self-report measures are a good indication for PTSD, but cannot replace clinical interviews that yield a diagnosis based on more detailed information, severity of symptoms and level of impairment in functioning.

With regard to permanent physical impairment, our results indicate a comparable outcome to the study of Zatzick et al. (2008), in which long-term physical impairment was associated with the occurrence of PTSD at 12 months follow-up. Furthermore, the outcome confirms the suggestion that continuing physical problems can contribute to ongoing psychological distress (Gillies et al. 2003).

With regard to acute pain, in our previous research (van Meijel et al. 2018), we found that severe acute pain was associated with the severity of posttraumatic stress 3 months later. These findings were not confirmed in our long-term results. A possible explanation is the use of dichotomous outcomes in the current study, instead of the continuously measured severity of symptoms in the previous study. The latter may be a more sensitive measure. Future research in larger samples may show whether acute pain is associated with longer-term PTSD or whether the long-term outcome is associated with different factors or a combination of factors.

The role of experiencing a new traumatic event is not clear. Delahanty and Nugent (2006) suggested that prior trauma history can increase vulnerability for PTSD in children and adults after experiencing a new traumatic event. In our study, children in the group with PTSD or PTSS reported more traumatic events in the past than those in the group without, and the percentage of children that experienced a new traumatic event was substantially higher in the affected group. However, the difference between the groups was not statistically significant.

For the three children in our sample who completed trauma-focused therapy, the therapy had a positive impact in the long term, although other factors may also have facilitated the reduction of PTSD symptoms. The group was too small to draw conclusions about an association between completing trauma-focused psychotherapy and long-term PTSD or PTSS. A recent systematic review and meta-analysis on trauma-focused psychotherapy emphasized the effectiveness of both Cognitive Behavior Therapy and EMDR in reducing posttraumatic stress symptoms (Khan et al. 2018), which is all the more reason to promote evidence-based trauma-focused psychotherapy.

Some children (or their parents on their behalf) do not seek treatment, even if they are advised to do so. Likewise, dropping out of therapy is a well-known problem (Stallard 2006). Possible barriers to seeking or accepting mental health treatment are low perceived need and a desire to handle the problems on one’s own (Andrade et al. 2014). Perceived stigma, time commitment or costs may also play a role in some families (Smith et al. 2018). Possible reasons for drop-out are perceived ineffectiveness of treatment and negative experiences with treatment providers (Andrade et al. 2014). With regard to injured children, the NICE guideline (NICE 2005) suggests that injured children who are still undergoing medical treatment, or who have to cope with permanent physical disability, probably judge these problems as more important than the need for treatment for psychological problems. Moreover, since avoidance is one of the symptoms of PTSD, it is likely that seeking and completing treatment will have to be promoted actively. Healthcare professionals can actively follow-up children with PTSD who miss scheduled appointments (NICE 2005). Furthermore, we will have to find effective ways to emphasize the importance of treatment, perhaps by exploring the use of peers and social media.

## Strengths and Limitations

A few limitations in this study need to be considered. First, since the follow-up assessment was not scheduled in the design of the initial study, the time between the first and the follow-up assessment ranged from 2 to 4 years. Therefore, although we conducted a long-term follow-up study, the findings may not be generalizable to other samples due to the resulting variability in children’s development and possible transitions in life. Second, follow-up participants reported fewer posttraumatic stress symptoms at T1 than non-participants did. If the loss to follow-up in the group with more symptoms would have been lower, it is likely that the prevalence of PTSD could have been higher. Third, in an ideal situation, we would have used logistic regression analysis to examine the association between multiple variables and PTSD and PTSS, and we would have accounted for the variance in the time between T1 and T2, which ranged between 2 and 4 years. However, the number of children with PTSD or PTSS in our sample was too low to perform this analysis (Peduzzi et al. 1996). Fourth, due to the range in ages of children, we used two different PTSS self-report measures, CRIES and IES-R. However, algorithms to transform raw scores into standardized scores are not available. Hence, we could not combine continuous data from these instruments and perform multivariate linear regression. Fifth, due to the acute situation after an accident, a retrospective pain rating was used. This increases the chance of unreliable pain ratings (Lewandowski et al. 2009; van Meijel et al. 2018). Sixth, since the T1 information was available to the interviewers, they were not blinded to diagnoses and scores. To account for the possible implications of this aspect of the study, the interpretation of the results was performed in cooperation with an independent clinical statistician.

The most important strength of this study is the much longer-term follow-up than in prior studies and the possibility to compare the results with short-term findings. Second, in addition to psychological aspects, we included acute pain and physical condition in our study. Third, we used parent and child-informed interviews as well as validated child questionnaires, thus increasing completeness and reliability of the information. Finally, although the sample size is relatively small, a 61% response for a long-term follow-up study is good. The results of our study can, therefore, make a valuable contribution to the overall knowledge of long-term consequences of accidental injury.

## Conclusions and Clinical Implications

Our findings show that the long-term prevalence of PTSD in children and adolescents following accidents is comparable to the short-term prevalence. Over the long term, PTSD was related to a new traumatic event or to the initial accident. In our study, a small number of children completed trauma-focused psychotherapy after the accident. At follow-up they were still free of posttraumatic stress symptoms, in contrast to those who did not complete psychotherapy. A substantial number of the participating children reported permanent physical impairment, ongoing physical problems and negative consequences on their education, social life and future plans. Our results revealed a substantial difference between children with and without PTSD regarding permanent physical impairment, indicating an association between the presence of PTSD and permanent physical impairment. Adolescence in combination with permanent impairment may have an influence on later PTSD as this can be a sensitive period in which this age group is modeling future plans. The consequence may be that adolescents are more at risk for long-term negative psychological outcome when permanent physical impairment negatively influences their future plans. Further research, preferably in a larger sample, is needed to test this hypothesis and other possible explanations regarding an association between permanent physical impairment and PTSD.

Our results have implications for clinical practice. To prevent long-term negative consequences of accidents, we recommend systematic monitoring—including screening—of injured children and their parents. The introduction of trauma-informed care can facilitate this process. Children with permanent physical impairment or ongoing physical problems may need special attention. For those who need it, we recommend active promotion of timely and appropriate evidence-based trauma-focused psychotherapy. Healthcare professionals should be aware of the importance of children completing their trauma-focused psychotherapy and should find ways to prevent drop-out.
